# Alone or in combination, hyaluronic acid and chondroitin sulfate alleviate ECM degradation in osteoarthritis by inhibiting the NF-κB pathway

**DOI:** 10.1186/s13018-024-05411-6

**Published:** 2025-01-04

**Authors:** Yiran Ma, Xin Yang, Min Jiang, Wangjuan Ye, Hong Qin, Songwen Tan

**Affiliations:** 1 Aunobel pty Ltd Nutrition and health research institute, Strathfield, 2135 NSW Australia; 2 Monash Suzhou Research Institute, Monash University, Suzhou, 215000 Jiangsu China

**Keywords:** Hyaluronic acid, Chondroitin sulfate, NF-κB, Osteoarthritis, Extracellular matrix

## Abstract

**Backgrounds:**

Osteoarthritis (OA) significantly impacts the elderly, leading to disability and decreased quality of life. While hyaluronic acid (HA) and chondroitin sulfate (CS) are recognized for their therapeutic potential in OA, their effects on extracellular matrix (ECM) degradation are not well understood. This study investigates the impact of HA and CS, individually and combined, on ECM degradation in OA and the underlying mechanisms.

**Methods:**

OA was modeled in rats through anterior cruciate ligament transection and in cells using IL-1β pretreatment. Treatments included HA and CS, alone or combined, with and without PMA (an NF-κB pathway activator). Cartilage tissue was analyzed using HE and Saffron O-fast green staining, with degradation assessed via the OARSI score. Inflammatory factors were measured by ELISA, and ECM-related proteins were detected by immunohistochemistry, immunofluorescence, and Western blotting. Chondrocyte viability was assessed using CCK8.

**Results:**

HA and CS treatments significantly reduced cartilage damage, decreased inflammatory factor release, alleviated ECM degradation, and inhibited NF-κB pathway activation compared to the OA group (*P* < 0.05). The combination of HA and CS further enhanced these therapeutic effects (*P* < 0.05). However, these benefits were reversed when PMA was introduced (*P* < 0.05).

**Conclusion:**

HA and CS, whether used alone or in combination, mitigate ECM degradation in osteoarthritis by inhibiting the NF-κB pathway, offering potential therapeutic benefits for OA management.

**Supplementary Information:**

The online version contains supplementary material available at 10.1186/s13018-024-05411-6.

## Introduction

Osteoarthritis presents as a chronic, progressive condition affecting bones and joints, distinguished by the deterioration and potential loss of articular cartilage. Additionally, it may present with comorbidities such as osteophyte formation and bursitis [1]. This disease has emerged as the most prevalent degenerative joint disorder worldwide, imposing a significant burden on the elderly population by leading to disability and compromising their overall quality of life [2]. While current treatments for osteoarthritis primarily target pain management through approaches such as physical therapy and pharmacological interventions [3], there’s an urgent need for therapeutic strategies that can slow the progression of the disease and improve bone and joint function.

In the pathogenesis of osteoarthritis, the NF-κB pathway emerges as a critical regulatory mechanism [4]. Its activation is intricately linked to various physiological and pathological processes, including inflammatory responses, apoptosis, and cell proliferation [5]. Research underscores the potential of HA and CS in mitigating the progression of osteoarthritis by modulating the NF-κB pathway. Zhang C et al. emphasized the therapeutic effects of HA in osteoarthritis by inhibiting NF-κB pathway activation [6]. Similarly, Vallières M et al. demonstrated CS can reduce NF-κB pathway activation and the nuclear translocation of chondrocytes, thereby offering therapeutic benefits in osteoarthritis [7]. Nonetheless, investigations into the individual or combined applications of HA and CS in modulating the NF-κB pathway remains relatively sparse.

Recent research underscores the pivotal significance of the NF-κB pathway in orchestrating ECM degradation in osteoarthritis [8, 9]. The pathophysiological cascade of osteoarthritis involves a complex interplay of various molecular, cellular, and biological mechanisms. Among these, the progressive erosion of cartilage ECM is recognized as a primary hallmark, characterized by reduced levels of aggrecan and collagen-II levels [10]. When the ECM functions optimally, joint movement experiences minimal friction, thereby protecting the osteoarticular joint from wear and tear. Conversely, disruption of the ECM renders joints susceptible to mechanical stress, promoting inflammation and cartilage damage [11]. However, the precise effects of HA and CS, whether administered individually or in combination, on ECM degradation in osteoarthritis via modulation of the NF-κB pathway requires further investigation.

Thus, this study aimed to investigate the influence of HA and CS, either individually or in combination, on the modulation of the NF-κB pathway in osteoarthritis and their impact on ECM degradation. By doing so, we seek to enhance our understanding of the pharmacological properties of HA and CS, while also generating novel insights for treatment strategies in osteoarthritis.

## Methods

### Reagents and instruments

Reagents: Hyaluronic acid (HA, 924474, Merck, Shanghai, China); Chondroitin sulfate (CS, Y0000593, Merck, Shanghai, China); Phorbol 12-myristate 13-acetate (PMA, P8139, Merck, Shanghai, China); Pentobarbital sodium (P3761, SIGMA, America); 10% Fetal bovine serum (FBS, 10100147 C, Thermo Fisher, Waltham, MA, USA); 1% penicillin-streptomycin (15140148; Thermo Fisher, Waltham, MA, USA); DMEM/F12 medium (D0697, Merck, Shanghai, China); TNF-α ELISA kit (A11534, Abclonal, Wuhan, China); IL-1β ELISA kit (A16288, Abclonal, Wuhan, China); IL-6 ELISA kit (A11114, Abclonal, Wuhan, China); Hematoxylin Eosin staining solution (G1005, Servicebio, Wuhan, China); Safranine O-Fast Green Stain Kit (R21845, Saint-Bio, Shanghai, China); CCK8 kit (C0037, Beyotime, Shanghai, China); Phosphate-buffer saline (PBS, P1020, Solarbio, Beijing, China).

Instruments: electrophoresis (WIX-EP600, wix, Beijing, China); Protein electrophoresis tank (WIX-miniPRO4, wix, Beijing, China); protein transfer groove (WIX-miniBLOT4, wix, Beijing, China); Chemiluminescent imaging system (SH-523, shenhuabio, Hanzhou, China); enzyme marker (Multiskan FC, ThermoFisher, Waltham, MA, USA); short-speed centrifuge (S1010E, Scilogex, C-6 Rocky Hill, CT, USA); High-speed freezing centrifuge (H1-16KR, kechengyiqi, Changsha, China); Decolourisation shaker (SLA-O3000-S, Scilogex, C-6 Rocky Hill, CT, USA); orthostatic microscope (MZ62, mshot, Guangzhou, China); fluorescence inverted microscope (MF52, mshot, Guangzhou, China); ultrapure water system (Smart-N15VF, healforce, Hong Kong, China); Electronic ice box (EICE4, eastwin, Suzhou, China); ice machine (IMS-20, csckdq, Changshu, China); Tissue Dehydrators (TS-C20, taiho, Hefei, China); Paraffin embedding machine (ES-300, bio-hisure, Jinhua, China); Paraffin Slicer (YD-335, zjyidi, Jinhua, China).

### Animal modeling and drug administration

Forty 6-week-old SD male rats (200–240 g) were purchased from Hunan Hu Jinda Laboratory Animal Co. Ltd, Production License No.: SCXK (Hunan) 2016-0002. Before the beginning of the experiment, the rats underwent a 3-day acclimatization period in a constant temperature (18–26 °C) and humidity (40–70% RH) environment. The study strictly followed the 3R principles and was approved by the Animal Committee (Ethics No. XZ202411). Rats were randomly divided into 6 groups, Sham group, OA group, OA + HA group, OA + CS group, OA + HA + CS group, and OA + HA + CS + PMA group, 5 rats in each group. anterior cruciate ligament transection (ACLT) was used in the OA, OA + HA, OA + CS, and OA + HA + CS + PMA groups to establish the OA rat model [12]. In the Sham group, the knee joint of the left hind limb was completely exposed and then the joint cavity and skin were sutured. The rats in the OA + HA group were intraperitoneally injected (IP) with 0.2mL HA (10 mg/ml dissolved in saline) [13], and the rats in the OA + CS group were IP with CS (100 mg/kg) [14]. The amount of IP of HA and CS in the OA + HA + CS group and the OA + HA + CS + PMA group was the same as that in the previous two groups, where the amount of HA and CS in the OA + + HA + CS + PMA group was the same as that in the previous two groups. In the OA + HA + CS + PMA group, PMA (10 mg/kg) was also injected, and 50 µL of sterile saline was injected intraperitoneally in the Sham group and the OA group as a control, and the frequency of injection was twice a week in both groups. The rats were anesthetized and executed by IP of sodium pentobarbital (140 mg/kg) at the end of the 8th week, and serum and bone tissues were collected.

### Cell culture and grouping

Neonatal (within 24–72 h of birth) male SD rats were purchased from Hunan Hu Jinda Laboratory Animal Co. Ltd, Production License No.: SCXK (Hunan) 2016-0002. This study strictly followed the 3R principle and was approved by the Animal Ethics Committee of Hunan Evidence-based Biotechnology Co., Ltd (Ethics No. XZ202411). Rats were anesthetized by IP of sodium pentobarbital (40 mg/kg) and then cervical vertebrae were dislocated to execute the rats. Primary rat knee chondrocytes were isolated, and the isolated chondrocytes were cultured in DMEM/F12 medium containing 10% FBS and 1% penicillin-streptomycin, and passaged at 37℃ with 5% CO2. The cultured 1–2 generations of chondrocytes were equally divided into 6 groups, Control group, OA group, OA + HA group, OA + CS group, OA + HA + CS group, and OA + HA + CS + PMA group. The chondrocytes of the five groups except the Control group were exposed to a medium containing IL-1β (20ng/mL) and incubated for 36 h to establish the OA cell model. The OA + HA group, the OA + CS group, the OA + HA + CS group, and the OA + HA + CS + PMA group were added to the medium with HA (20 mg/mL), CS (100 mg/mL), and PMA ( 10 mg/mL), and equal amounts of DMEM/F12 medium were added to the Control and OA groups.

### HE staining

The rat bone tissue was fixed in 10% neutral buffered formalin, dehydrated through a graded series of ethanol, cleared with xylene, and embedded in paraffin for sectioning. The paraffin sections of bone tissue were deparaffinized with xylene and rehydrated through graded ethanol to water. Hematoxylin and eosin (H&E) staining was performed, followed by clearing in xylene and dehydration through a graded ethanol series. Finally, the sections were mounted with neutral resin and observed under a standard optical microscope for imaging.

### Saffron O-fast green staining

The bone tissue was decalcified by soaking in 5% calcium acetate for 1 day, followed by paraffin embedding to prepare paraffin sections. The paraffin sections of bone tissue were deparaffinized with xylene and rehydrated through graded ethanol to water. Iron hematoxylin staining was followed by an acidic differentiation solution, the solid green staining solution was added to fix the green, the sections were washed with a weak acid solution, followed by immersion staining using senna staining solution, and then dehydrated in xylene and ethanol sequentially, and the sections were sealed and then viewed under an ordinary light microscope.

### OARSI score

Specimens to be analyzed were coronal sectioned after routine embedding, stained, and then observed and scored microscopically. Two main aspects were included: articular cartilage damage and osteochondrogenesis. The articular cartilage was scored on the following scale: 0 = normal; 0.5 = very minimal degeneration. Small loss of blue (or other cationic dye) staining (loss of proteoglycans), but no structural changes; 1 = very small degeneration: small surface abrasion and fibrosis below the cartilage surface are seen, with no loss of chondrocytes or cartilage matrix, and the area of damage is less than 5% of the total area; 2 = mild degeneration: the damage is vertically and below the surface of the cartilage, but seldom extends deeper into the cartilage layer. There is partial loss of cartilage surface matrix or localized areas of chondrocyte/proteoglycan loss are seen, but there is still good collagen preservation, and the area of cartilage damage is about 5–10% of the total cartilage surface; 3 = Moderate Degeneration: the injury extends vertically and downward into the calcarine layer of cartilage, with localized areas of chondrocyte/proteoglycan loss, and about 10–24% of articular cartilage is involved; 4 = Marked Degeneration: Vertical fissure/damage extending below the calcified cartilage, more than 25–50% of the articular surface of the cartilage, or loss of localized areas of chondrocytes/proteoglycans, involving 25–50% of the thickness of the articular cartilage; 5 = Severe Degeneration: vertical fissure/damage extending below the calcified cartilage of the articular, with 50–75% of the surface of the cartilage compromised or localized areas of chondrocytes/proteoglycans missing, with approximately 50–75% of cartilage thickness is compromised; 6 = very severe degeneration: vertical fissures/destruction of cartilage extending below the calcified cartilage, greater than 75% of the articular surface is compromised, and there may be only small areas of cell-free collagen remaining. Osteochondral scoring and measurement: largest osteochondral (tibial or femoral) measured using an eyepiece, 1 = small (< 150 μm); 2 = medium (151 to 300 μm); 3 = large (> 300 μm).

### Immunohistochemistry

The paraffin sections of cartilage tissue were deparaffinized with xylene, rehydrated through graded ethanol to water, and underwent heat-induced antigen retrieval. Endogenous enzymes were eliminated using a 3% aqueous hydrogen peroxide solution. and sealed in a PBS solution containing 10% BSA (JK-E1344, Jkbio, Shanghai, China) for 1 h. Subsequently, the sections were exposed to specific primary antibodies Collagen-II (ab307674, 1:1000, abcam, Shanghai, China) and Aggrecan (MA3-16888, 1:1000, ThermoFisher, Waltham, MA, USA), and incubated overnight at 4 °C before coupling with HRP of the primary antibody secondary antibody was incubated for 30 min at room temperature. Color development was performed using DAB colour development kit (G1212, Servicebio, Wuhan, China), followed by counterstaining with hematoxylin. Imaging was captured using digital confocal microscopy.

### ELISA

Chondrocytes were centrifuged and the supernatant was collected, and cartilage tissue was added to RIPA buffer to extract tissue stock. The supernatant and tissue stock were collected and the levels of TNF-α, IL-6, and IL-1β in chondrocytes and cartilage tissues were determined using ELISA kits.

### Western blot

Total protein lysates within chondrocytes and cartilage tissues were obtained using RIPA lysate (P0013B, Beyotime, Shanghai, China) and protein concentrations were determined using the BCA protein quantification kit (P0009, Beyotime, Shanghai China), followed by upsampling of protein samples onto polyacrylamide gels (SDS-PAGE, G2003-50T, Servicebio, Wuhan, China) for electrophoretic separation. After transferring the samples to a PVDF membrane (WJ001S, epizyme, Shanghai China), the membrane was closed with 5% skimmed milk powder, and the samples were incubated with anti-IκBα (A11168, 1:1000, Abclonal, Wuhan, China), p-IκBα (AP0614, 1:1000, Abclonal, Wuhan, China), p65 (A22684, 1:1000, Abclonal, Wuhan, China), and p-65 (AP0123, 1:1000, Abclonal, Wuhan, China). Shanghai China), Collagen-II (A24863, 1:1000, Abclonal, Wuhan, China), Aggrecan (MA3-16888, 1:1000, ThermoFisher, Waltham, MA, USA) and GAPDH (AC001, 1:1000, Abclonal, Wuhan, China) primary antibodies were incubated at 4 °C overnight. Then it was incubated with HRP-coupled secondary antibody (1:2000, Proteintech) at room temperature for 2 h. It was washed with TBST solution for 5 min and developed using BeyoECL Star (G2020, Servicebio, Wuhan, China) assay for 30 s. The gray values of the bands were analyzed using ImageJ software (V1.8.0.112, NIH, Madison, WI, USA).

### CCK-8

Chondrocytes (1 × 104/well) from each group were inoculated into 96-well plates separately, and CCK-8 reagent (10 µL) was added to the inoculated cells in each well, and then incubated for 2 h at 37 °C with 5% CO_2_. The absorbance of each well was measured at 450 nm using a microplate spectrophotometer, and the absorbance values were positively correlated with the proliferative activity of chondrocytes.

### Immunofluorescence

Chondrocytes were fixed in 4% paraformaldehyde for 15 min and washed three times with PBS. The chondrocytes were subsequently closed using normal goat serum (S9070, solarbio, Beijing, China) at room temperature for 30 min. Chondrocytes were incubated with primary antibodies against Aggrecan (MA3-16888, 1:500, Thermo Fisher, Waltham, MA, USA) and Collagen-II (ab307674, 1:500, abcam, Shanghai, China) for 24 h at 4 °C, and then incubated with secondary antibodies for 1 h at room temperature. After three rinses of PBS, the membrane was observed under a fluorescence microscope and images were captured. Calculate the fluorescence positivity rate using ImageJ software (V1.8.0.112, NIH, Madison, WI, USA).

### Statistical analysis

Data were expressed as mean ± standard deviation. For data obeying normal distribution, differences were tested using analysis of variance one-way analysis of variance (ANOVA), and multiple comparisons were performed using Bonferroni’s test. Statistical analyses were performed using SPSS 22.0 (IBM, Corp., Armonk, NY, USA) software, and data were plotted using GraphPad Prism (v. 9.0, La Jolla, CA), and differences were considered significant when *P* < 0.05.

## Results

### HA and CS, alone or in combination, effectively protect cartilage tissue from damage in OA rats

**Fig. 1:**
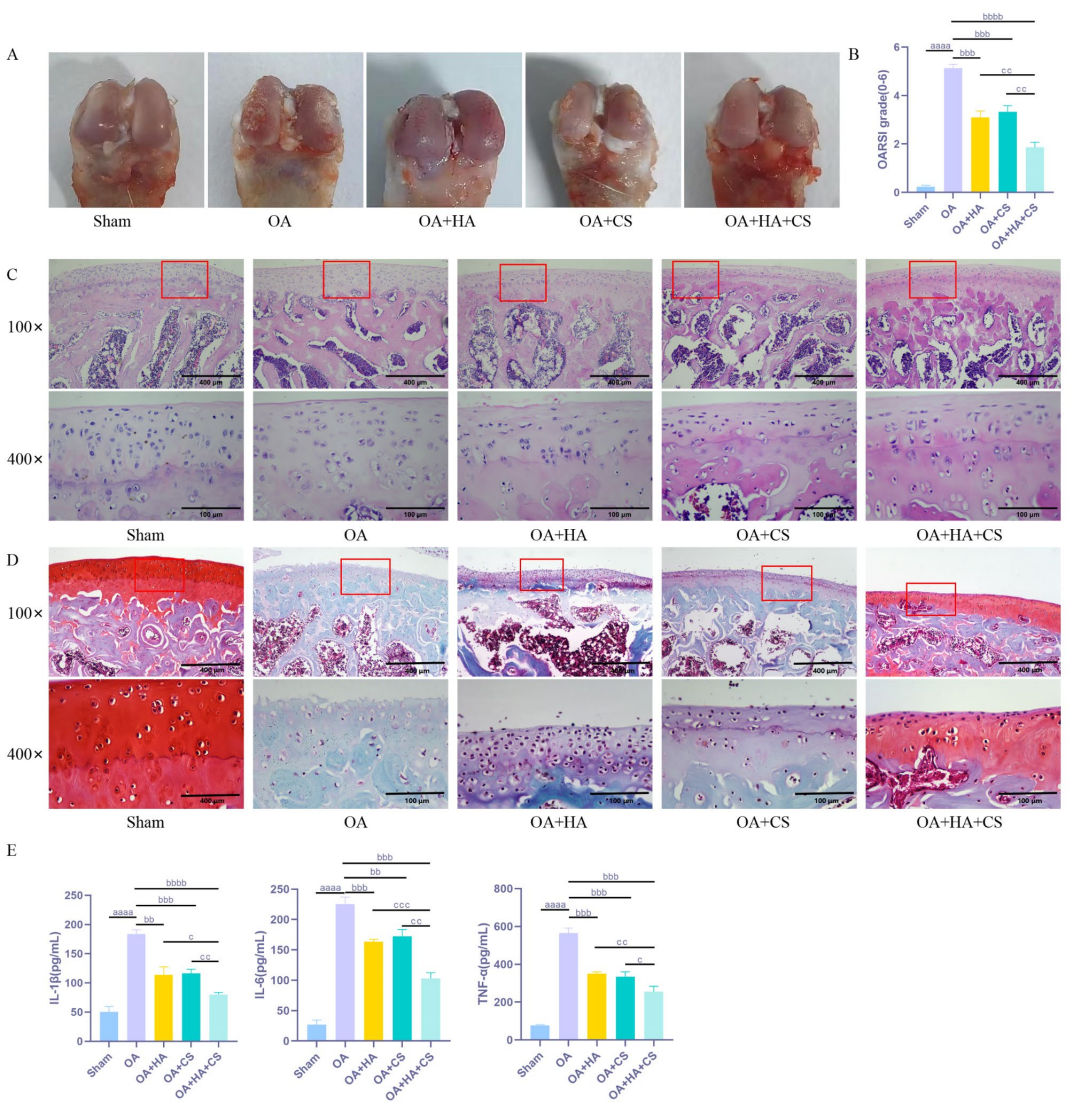
HA and CS, alone or in combination, effectively protect cartilage tissue from damage in OA rats. (**A**) rat bone and joint; (**B**) OARSI score to assess the degree of cartilage degeneration in rats; (**C**) HE staining to assess the extent of bone tissue damage in rats; (**D**) Saffron O-fast green staining to assess the extent of bone tissue damage in rats (100×, Scale = 400 μm; 400×, Scale = 100 μm); (**E**) Expression of inflammatory factors (IL-1β, IL-6, TNF-α) in cartilage tissues detected by ELISA. ^aaaa^*P* < 0.0001 vs. Sham, ^bb^*P* < 0.01 vs. OA, ^bbb^*P* < 0.001 vs. OA, ^bbbb^*P* < 0.0001 vs. OA, ^c^*P* < 0.05 vs. OA + HA + CS, ^cc^*P* < 0.01 vs. OA + HA + CS, ^ccc^*P* < 0.001 vs. OA + HA + CS. *N* = 5

In order to investigate the protective effects of HA and CS individually and in combination on the cartilage tissue of OA rats, the present study aimed to observe the degree of damage to the cartilage tissue of OA rats through HE staining and Senna-solid green staining. Additionally, the study evaluated the degree of cartilage degradation using the OARSI scoring system and measured the expression levels of inflammatory factors (TNF-α, IL-6, and IL-1β) in the cartilage tissue via ELISA. The staining results (Fig. 1) indicated that the rats in the Sham group exhibited normal cartilage thickness, a smooth cartilage surface, normal chondrocyte morphology, a uniform red cartilage matrix, and a sharp contrast between cartilage and bone tissues. In contrast, the rats in the OA group displayed cartilage thinning, surface defects, a markedly reduced and more flocculent arrangement of chondrocytes, disrupted tidemarks, a reduction in cartilage tissue area, and osteoid formation. The extent of cartilage damage in OA rats was mitigated by the administration of HA and CS alone, and significantly enhanced by the combined use of HA and CS. According to the OARSI score, the OA group exhibited significantly higher scores compared to the Sham group. Conversely, treatment with HA and CS, either alone or in combination, resulted in significantly lower scores than those observed in the OA group. Notably, the OA + HA + CS group demonstrated the lowest scores among all treatment groups (*P* < 0.05). ELISA analysis revealed elevated levels of inflammatory factors in the OA group relative to the Sham group. In contrast, the treatment groups exhibited markedly reduced expression levels of inflammatory factors compared to the OA group, with the combined treatment group showing the lowest levels (*P* < 0.05). These findings collectively suggest that both HA and CS provide protective effects against cartilage damage in OA rats, with the combined treatment demonstrating superior efficacy compared to individual treatments.

### HA and CS, alone or in combination, effectively inhibit ECM degradation in cartilage tissue of OA rats

**Fig. 2:**
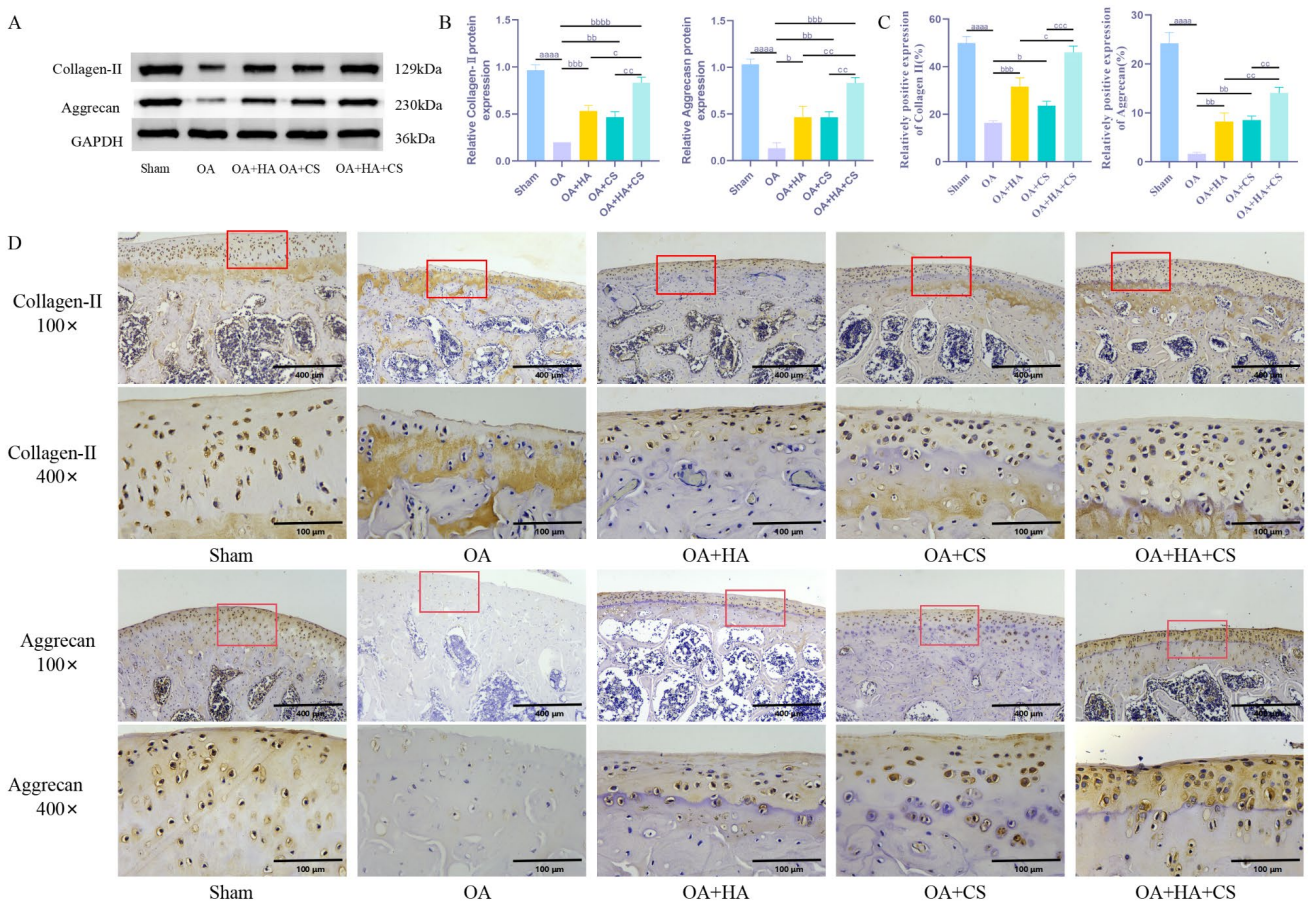
HA and CS, alone or in combination, effectively inhibit ECM degradation in cartilage tissue of OA rats. (**A-B**) WB detection of ECM-related proteins (Collagen-II, Aggrecan) in cartilage tissue of rats; (**C-D**) Immunohistochemical detection of ECM-related proteins (Collagen-II, Aggrecan) in cartilage tissue of rats (100×, Scale = 400 μm; 400×, Scale = 100 μm). ^aaaa^*P* < 0.0001 vs. Sham, ^b^*P* < 0.05 vs. OA, ^bb^*P* < 0.01 vs. OA, ^bbb^*P* < 0.001 vs. OA, ^bbbb^*P* < 0.0001 vs. OA, ^c^*P* < 0.05 vs. OA + HA + CS, ^cc^*P* < 0.01 vs. OA + HA + CS, ^ccc^*P* < 0.001 vs. OA + HA + CS. *N* = 5

In osteoarthritis, the progressive degeneration of cartilage ECM is recognized as a hallmark feature. To investigate the effects of HA and CS, either individually or in combination, on the ECM in cartilage tissues of OA rats, levels of Collagen-II and Aggrecan were assessed through WB and immunohistochemistry. The findings (Fig. 2) revealed significantly decreased levels of Aggrecan and Collagen-II in the OA group compared to the Sham group. Conversely, the OA + HA and OA + CS groups exhibited elevated levels of Aggrecan and Collagen-II in comparison to the OA group. Notably, the combined treatment group demonstrated even higher Collagen-II content than either of the individual treatment groups (*P* < 0.05). These results suggest that both HA and CS effectively attenuate ECM degradation in the cartilage tissues of OA rats, with the combined treatment displaying superior efficacy over the individual treatments.

### HA and CS, alone or in combination, contribute to slowing down the degradation of ECM in cartilage tissues of OA rats by inhibiting the NF-κB pathway

**Fig. 3:**
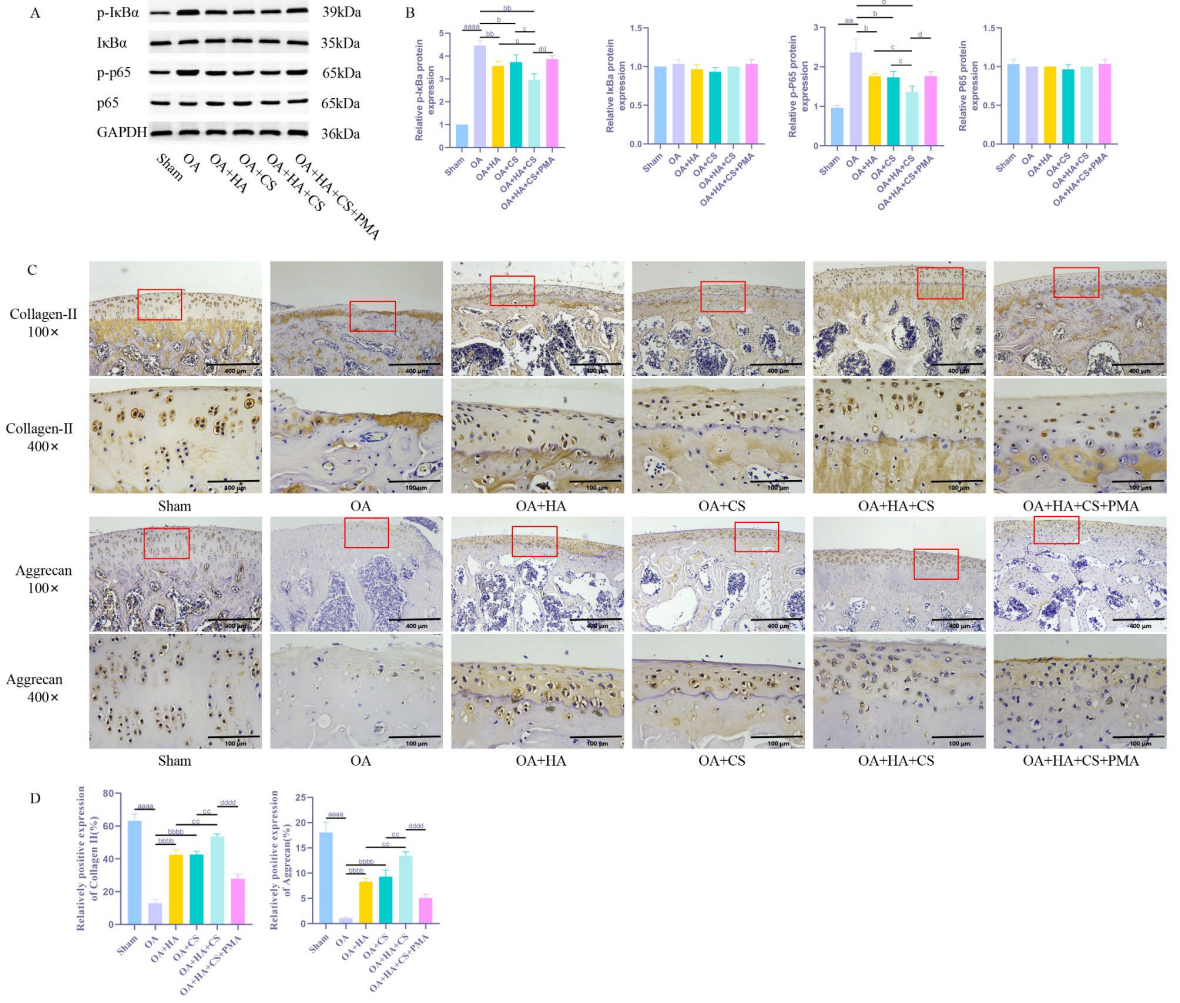
HA and CS, alone or in combination, contribute to slowing down the degradation of ECM in cartilage tissues of OA rats by inhibiting the NF-κB pathway. (**A**) WB detection of IκBα, p-IκBα, p65 and p-p65 expression in rat cartilage tissue; (**B**) Immunohistochemical detection of ECM-related proteins (Collagen-II, Aggrecan) in cartilage tissue of rats (100×, Scale = 400 μm; 400×, Scale = 100 μm). ^aa^*P* < 0.01 vs. Sham, ^aaaa^*P* < 0.0001 vs. Sham, ^b^*P* < 0.05 vs. OA, ^bb^*P* < 0.01 vs. OA, ^bbbb^*P* < 0.0001 vs. OA, ^c^*P* < 0.05 vs. OA + HA + CS, ^cc^*P* < 0.01 vs. OA + HA + CS, ^d^*P* < 0.05 vs. OA + HA + CS + PMA, ^dd^*P* < 0.05 vs. OA + HA + CS + PMA, ^dddd^*P* < 0.0001 vs. OA + HA + CS + PMA. *N* = 5

In subsequent steps, we administered an NF-κB pathway activator (PMA) to the combined treatment group. Using WB and immunohistochemistry, we investigated NF-κB pathway proteins and ECM-associated proteins to elucidate the underlying mechanism by which the HA and CS combination alleviates ECM degradation in OA rat cartilage tissues. Our findings (Fig. 3) revealed a significant reduction in the levels of p-p65 and p-IκBα in the OA + HA and OA + CS groups compared to the OA group (*P* < 0.05). Moreover, the OA + HA + CS group exhibited an even more pronounced decrease in p-p65 and p-IκBα levels compared to the individual treatment groups following combination treatment (*P* < 0.05). Additionally, upon activation of the NF-κB pathway with PMA, the OA + HA + CS + PMA group demonstrated significantly lower levels of Aggrecan and Collagen-II compared to the OA + HA + CS group (*P* < 0.05). These findings collectively suggest that the combined application of HA and CS effectively delays ECM degradation in OA rat cartilage tissues by suppressing the activation of the NF-κB pathway.

### HA and CS, alone or in combination, help promote chondrocyte viability and slow down the degradation of ECM in chondrocytes

**Fig. 4:**
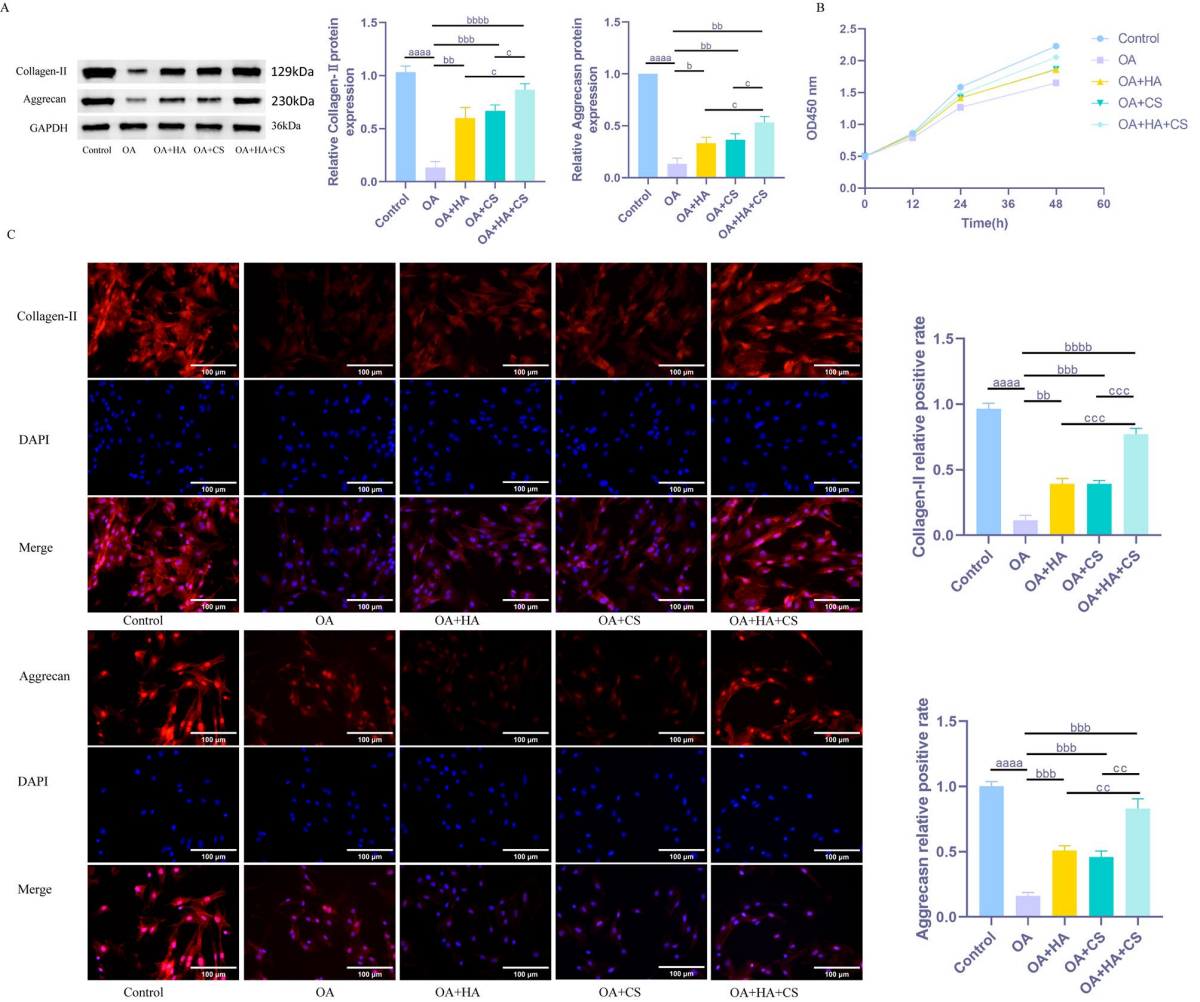
HA and CS, alone or in combination, help promote chondrocyte viability and slow down the degradation of ECM in chondrocytes. (**A**) WB detection of fluorescence intensity of ECM-related proteins (Collagen-II, Aggrecan) in chondrocytes; (**B**) CCK-8 detects rat chondrocyte viability; (**C**) Fluorescence intensity of ECM-related proteins (Collagen-II, Aggrecan) in chondrocytes detected by immunofluorescence. ^aaaa^*P* < 0.0001 vs. Sham, ^b^*P* < 0.05 vs. OA, ^bb^*P* < 0.01 vs. OA, ^bbb^*P* < 0.001 vs. OA, ^bbbb^*P* < 0.0001 vs. OA, ^c^*P* < 0.05 vs. OA + HA + CS, ^cc^*P* < 0.01 vs. OA + HA + CS, ^ccc^*P* < 0.001 vs. OA + HA + CS. *N* = 5

Subsequently, in vitro experiments were conducted to examine the effects of HA and chondroitin sulfate, either individually or in combination, on chondrocytes. The findings (Fig. 4) revealed a significant reduction in chondrocyte vitality, as well as Aggrecan and Collagen-II levels, in the OA group compared to the Control group (*P* < 0.05). In contrast, treatment with HA and CS, whether administered individually or in combination, resulted in a notable increase in chondrocyte vitality and in Aggrecan and Collagen-II levels compared to the OA group (*P* < 0.05). Moreover, the combined OA + HA + CS treatment exhibited significantly enhanced chondrocyte vitality and elevated levels of Aggrecan and Collagen-II compared to the individual treatments (*P* < 0.05). These findings collectively indicate that both HA and CS promote chondrocyte vitality and inhibit ECM degradation in chondrocytes, with the combined treatment demonstrating superior efficacy over the individual therapies.

### HA and CS, alone or in combination, can reduce ECM degradation in chondrocytes by inhibiting the NF-κB pathway

**Fig. 5:**
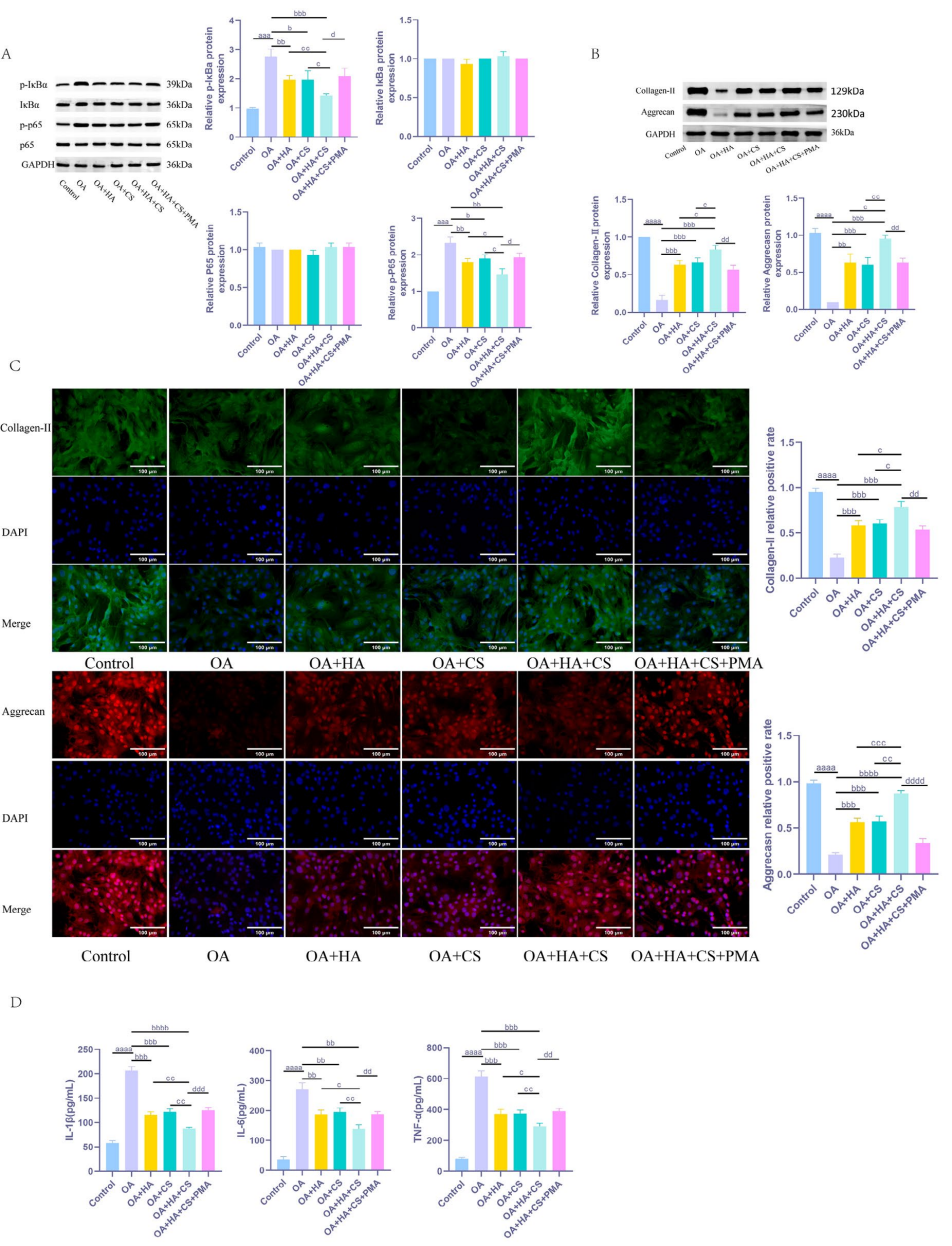
HA and CS, alone or in combination, can reduce ECM degradation in chondrocytes by inhibiting the NF-κB pathway. (**A**) WB detection of NF-κB pathway-related proteins (IκBα, p-IκBα, p65, p-p65) expression in chondrocytes; (**B**) WB detection of fluorescence intensity of ECM-related proteins (Collagen-II, Aggrecan) in chondrocytes; (**C**) Fluorescence intensity of ECM-related proteins (Collagen-II, Aggrecan) in chondrocytes detected by immunofluorescence; (**D**) Expression levels of IL-1β, IL-6 and TNF-α in chondrocytes detected by ELISA. ^aaa^*P* < 0.001 vs. Sham, ^aaaa^*P* < 0.0001 vs. Sham, ^b^*P* < 0.05 vs. OA, ^bb^*P* < 0.01 vs. OA, ^bbb^*P* < 0.001 vs. OA, ^bbbb^*P* < 0.0001 vs. OA, ^c^*P* < 0.05 vs. OA + HA + CS, ^cc^*P* < 0.01 vs. OA + HA + CS, ^ccc^*P* < 0.001 vs. OA + HA + CS, ^d^*P* < 0.05 vs. OA + HA + CS + PMA, ^dd^*P* < 0.01 vs. OA + HA + CS + PMA, ^ddd^*P* < 0.001 vs. OA + HA + CS + PMA, ^dddd^*P* < 0.0001 vs. OA + HA + CS + PMA. *N* = 5

In conclusion, this study employed an NF-κB pathway activator (PMA) in the combined treatment group to investigate how the combination of HA and CS affects the expression of inflammatory factors in chondrocytes and modulates ECM degradation. Our results (Fig. 5) demonstrated that, compared to the OA group, the expression of p-IκBα and p-p65 was significantly reduced in chondrocytes from the OA + HA and OA + CS groups, which was associated with decreased levels of IL-1β, IL-6, and TNF-α, alongside elevated levels of Aggrecan and Collagen-II (*P* < 0.05). Furthermore, in comparison to the individual treatment groups, the OA + HA + CS group exhibited greater reductions in p-IκBα and p-p65 expression, as well as more pronounced decreases in IL-1β, IL-6, and TNF-α levels, and higher levels of Aggrecan and Collagen-II (*P* < 0.05). Conversely, following NF-κB pathway activation, the OA + HA + CS + PMA group showed significantly increased expression levels of p-IκBα and p-p65, elevated levels of IL-1β, IL-6, and TNF-α, and decreased levels of Aggrecan and Collagen-II compared to the OA + HA + CS group (*P* < 0.05). These findings collectively indicate that the combination of HA and CS effectively mitigates the expression of inflammatory factors in OA chondrocytes and reduces ECM degradation by inhibiting NF-κB pathway activation.

## Discussion

Osteoarthritis, a chronic joint condition, is characterized by cartilage degeneration and tissue alterations, resulting in pain and impaired mobility [15]. HA present in joint fluid enhances lubrication, thereby improving joint function and alleviating discomfort [16]. CS provides protective effects on cartilage and possesses anti-inflammatory and antioxidant properties [17]. Our study, employing an ACLT-induced rat osteoarthritis model, demonstrated that HA and CS, either individually or in combination, significantly reduced inflammatory factors in rat cartilage tissues and conferred protection against damage. Notably, the combined treatment exhibited superior anti-inflammatory and cartilage-protective effects.

The development of osteoarthritis involves complex interactions among various cellular, molecular, and biological processes. Notably, dysregulated ECM degradation emerges as a significant pathological hallmark of the disease [10]. Comprising a diverse array of proteins, polysaccharides, and bioactive molecules such as aggrecan and collagen-II, the ECM plays a crucial role in maintaining the integrity of cartilage tissue [18]. As osteoarthritis progression, ECM degradation accelerates, leading to cartilage breakdown, compromised joint function, and increased pain levels [16]. This study further investigated, through both in vivo and in vitro experiments, how HA and CS, either individually or in combination, effectively enhanced chondrocyte viability and mitigated ECM degradation. Notably, the combined treatment demonstrated superior efficacy compared to individual interventions.

The abnormal degradation of the extracellular matrix (ECM) is closely associated with the activation of the NF-κB pathway [19, 20]. The NF-κB pathway is activated in osteoarthritic (OA) chondrocytes [21], promoting the secretion of pro-inflammatory cytokines such as TNF-α, IL-1β, and IL-6 [22]. These cytokines can directly stimulate chondrocytes to express and secrete matrix metalloproteinases (e.g., MMP-3, MMP-13) and ADAMTS proteases (e.g., ADAMTS-4, ADAMTS-5) [23, 24]. These enzymes serve as the primary degraders of aggrecan and collagen-II, cleaving their core proteins and resulting in significant degradation of these key ECM components [25, 26]. Additionally, these cytokines can directly inhibit the gene expression of aggrecan and collagen-II by regulating the activity of transcription factors such as SOX9 [27, 28]. In this study, we also observed that in the osteoarthritis model, the levels of TNF-α, IL-1β, and IL-6 were significantly elevated. Hyaluronic acid and chondroitin sulfate, whether administered alone or in combination, effectively reduced the levels of TNF-α, IL-1β, and IL-6, with the combination treatment demonstrating a superior effect compared to individual therapies. However, the molecular mechanisms underlying these effects remain to be elucidated.

HA and CS exhibit significant potential in modulating the NF-κB pathway [6, 7]. In this study, we investigated the influence of the NF-κB pathway on ECM degradation. This pathway serves as a crucial regulator linked to various processes, including inflammation and cellular behavior [5]. Previous research has underscored its role in the progression of osteoarthritis, where abnormal activation leads to adverse effects on cartilage [29]. Consequently, targeting this pathway may offer promising avenues for osteoarthritis treatment. Our experiments, conducted both in vivo and in vitro, demonstrated the effectiveness of HA and CS, either alone or in combination, in inhibiting NF-κB pathway activation. Furthermore, we observed that PMA, an NF-κB pathway activator, reversed the effects of the combined HA and CS treatment in our study. This suggests that hyaluronic acid and chondroitin sulfate, whether utilized individually or in tandem, can inhibit the secretion of inflammatory cytokines by suppressing the activation of the NF-κB pathway, thereby reducing chondrocyte inflammation and ECM degradation. However, it is important to note that additional molecular pathways may also contribute to the regulating of ECM degradation in osteoarthritis, and further research could yield a more comprehensive understanding.

In summary, this investigation presents innovative strategies for the treatment of OA. The combined application of HA and CS demonstrates significant potential in alleviating chondrocyte inflammation and ECM degradation by targeting the NF-κB pathway, thereby offering renewed hope for OA patients. However, further studies are warranted to validate these findings and refine therapeutic approaches for enhanced clinical efficacy.

## Conclusion

**Fig. 6:**
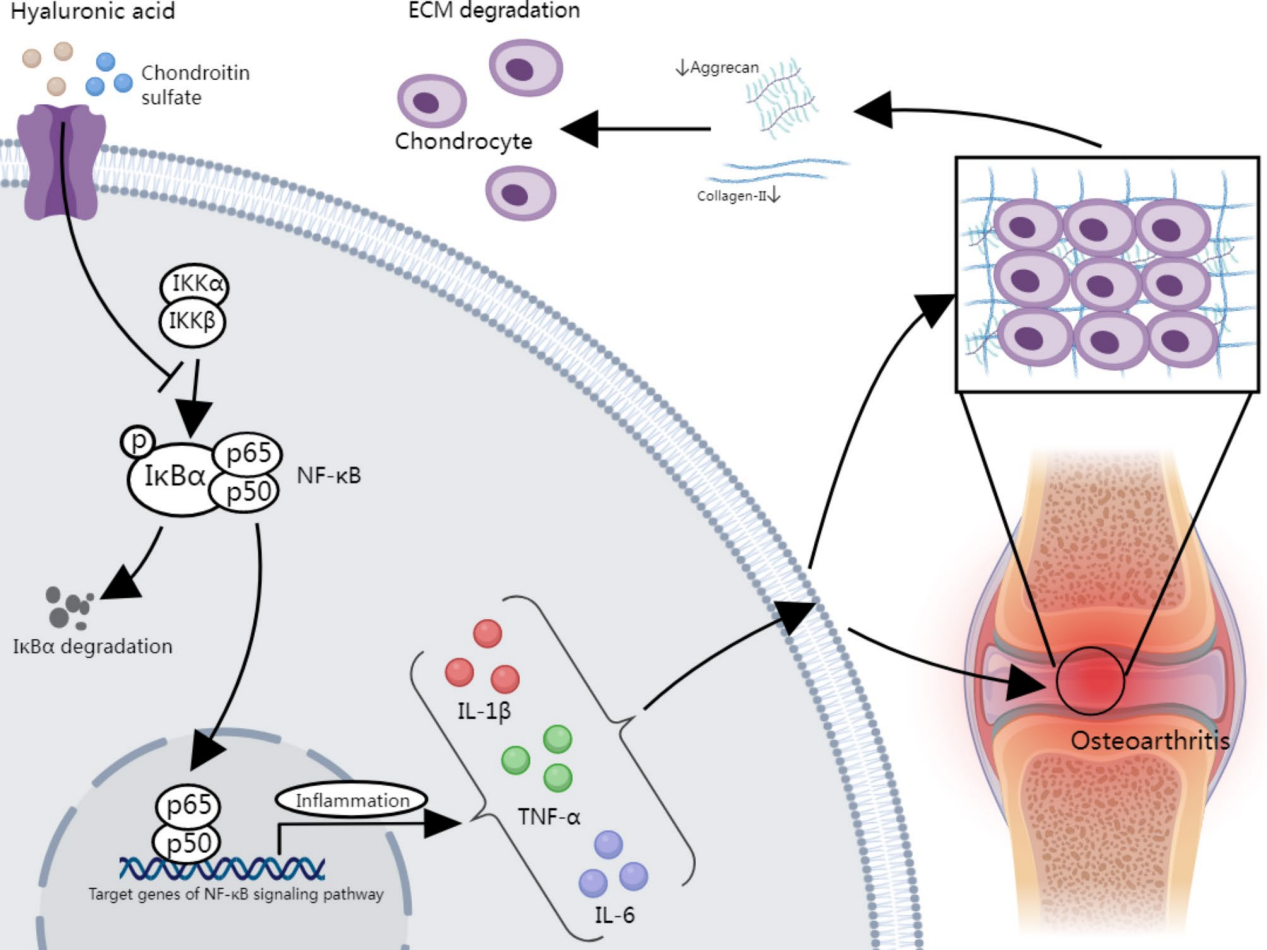
Mechanism by which hyaluronic acid and chondroitin sulfate, individually or in combination, alleviate ECM degradation in osteoarthritis by inhibiting the NF-κB pathway. Hyaluronic acid and chondroitin sulfate reduce the secretion of pro-inflammatory cytokines TNF-α, IL-6, and IL-1β by suppressing the activation of the NF-κB pathway, thereby alleviating inflammation and preventing the reduction of chondrocyte EMT-related proteins Aggrecan and Collagen-II. This, in turn, mitigates the abnormal degradation of the chondrocyte ECM

In summary, our results suggest that HA and CS, alone or in combination, effectively suppress the NF-κB pathway, slow down the process of ECM degradation in cartilage tissue, and slow down the progression of osteoarthritis in rats, which further provides a possible therapeutic option for osteoarthritis. The relevant mechanism is shown in Fig. 6.

## Electronic supplementary material

Below is the link to the electronic supplementary material.

Supplementary Material 1

Supplementary Material 2

## Data Availability

No datasets were generated or analysed during the current study.
