# Supplementary material for: Alone or in combination, hyaluronic acid and chondroitin sulfate alleviate ECM degradation in osteoarthritis by inhibiting the NF-κB pathway
Source: J Orthop Surg Res. 2025 Jan 4;20:11. doi: 10.1186/s13018-024-05411-6 (PMC11699666; doi:10.1186/s13018-024-05411-6)
Supplement: Supplementary file 2 — Supplementary Material 2 [file 13018_2024_5411_MOESM2_ESM.pdf]

Approval of Animal Experimental Ethical Inspection

No.XZ202411

**entry name**	Alone or in Combination, Hyaluronic Acid and Chondroitin Sulfate Alleviate ECM Degradation in Osteoarthritis by Inhibiting the NF-κB Pathway						
**name**	Songwen Tan	**Gender**	Male	**Education**	Ph.D	**Title**	None

Summary of research content:

OA was modeled in rats through anterior cruciate ligament transection and in cells using IL-1β pretreatment. Treatments included HA and CS, alone or combined, with and without PMA (an NF-κB pathway activator). Cartilage tissue was analyzed using HE and Saffron O-fast green staining, with degradation assessed via the OARSI score. Inflammatory factors were measured by ELISA, and ECM-related proteins were detected by immunohistochemistry, immunofluorescence, and Western blotting. Chondrocyte viability was assessed using CCK8

The applicant (project leader) promises:

The above information is true. If approved, I will conduct the research in strict accordance with the provided scheme and comply with the relevant regulations of the Animal Ethics Committee of Hunan Evidence-based Biotechnology Co., Ltd.

Signature of applicant (project leader): Songwen Tan

Date: October 29, 2023

Approval opinions of the Ethics Committee:

Agree.

湖南循证生物科技有限公司
动物实验伦理委员会

Ethics Committee (seal)

Date: November 5, 2024
